# Quantitative Proteomics Analysis Reveals the Min System of *Escherichia coli* Modulates Reversible Protein Association with the Inner Membrane* [Fn FN2]

**DOI:** 10.1074/mcp.M115.053603

**Published:** 2016-02-17

**Authors:** Hsiao-Lin Lee, I-Chen Chiang, Suh-Yuen Liang, Der-Yen Lee, Geen-Dong Chang, Kwan-Yu Wang, Shu-Yu Lin, Yu-Ling Shih

**Affiliations:** From the ‡Institute of Biological Chemistry, Academia Sinica, Taipei 115, Taiwan;; §Institute of Biochemical Sciences, College of Life Science, National Taiwan University, Taipei 106, Taiwan;; ¶Department of Microbiology, College of Medicine, National Taiwan University, Taipei 100, Taiwan;; ‖Department of Applied Chemistry, National Chiayi University, Chiayi 600, Taiwan

## Abstract

The Min system of *Escherichia coli* mediates placement of the division septum at the midcell. It oscillates from pole to pole to establish a concentration gradient of the division inhibition that is high at the poles but low at the midcell; the cell middle thereby becomes the most favorable site for division. Although Min oscillation is well studied from molecular and biophysical perspectives, it is still an enigma as to whether such a continuous, energy-consuming, and organized movement of the Min proteins would affect cellular processes other than the division site selection. To tackle this question, we compared the inner membrane proteome of the wild-type and Δ*min* strains using a quantitative approach. Forty proteins that showed differential abundance on the inner membrane of the mutant cells were identified and defined as proteins of interest (POIs). More than half of the POIs were peripheral membrane proteins, suggesting that the Min system affects mainly reversible protein association with the inner membrane. In addition, 6 out of 10 selected POIs directly interacted with at least one of the Min proteins, confirming the correlation between POIs and the Min system.

Further analysis revealed a functional relationship between metabolism and the Min system. Metabolic enzymes accounted for 45% of the POIs, and there was a change of metabolites in the related reactions. We hypothesize that the Min system could alter the membrane location of proteins to modulate their enzymatic activity. Thus, the metabolic modulation in the Δ*min* mutant is likely an adaptive phenotype in cells of abnormal size and chromosome number due to an imbalanced abundance of proteins on the inner membrane. Taken together, the current work reports novel interactions of the Min system and reveals a global physiological impact of the Min system in addition to the division site placement.

The rod-shaped bacterium *Escherichia coli* ^1^ undergoes binary fission resulting in two equal-sized daughter cells. The division site selection in *E. coli* is determined by both nucleoid occlusion and the Min system. While the nucleoid occlusion mechanism prevents division at the cylindrical part of the cell where the nucleoid mass is high, the Min system blocks division at the poles. The coordination of the two systems controls the precision of the division site placement (1). The Min system consists of three proteins: MinC, MinD, and MinE (2). MinC is a cell division inhibitor that interferes with the formation of the FtsZ ring, which further blocks formation of the division septum (3). MinD and MinE function together to form a membrane-associated oscillator that travels between the two cell poles (4–6). During oscillation, ATP hydrolysis in MinD, which is stimulated by interaction with MinE, is critical for driving oscillation of MinDE (7). MinC interacts with MinD and is dynamically positioned at the poles by MinDE, where it prevents aberrant polar division (Figs. 1*A* and 1*B*) (4, 6, 8). Although the molecular mechanism underlying oscillation of the Min proteins has been well studied (9), an unanswered question is whether this membrane-associated, energy-consuming, and rapidly oscillating MinDE machine affects additional physiological processes besides the division site selection.

**Fig. 1.**
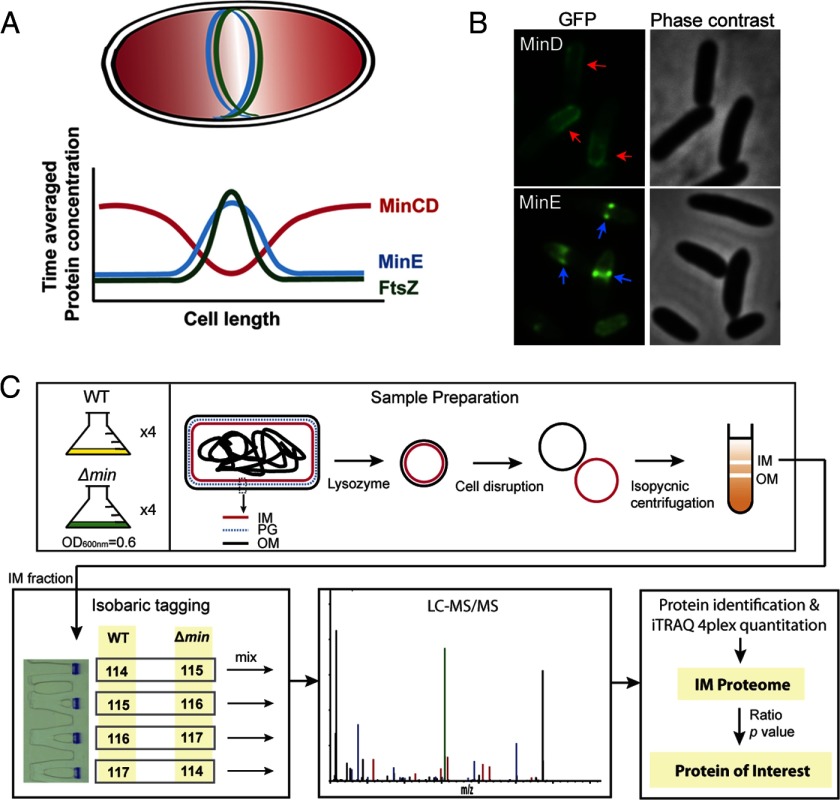
**Background and experimental procedures of the quantitative proteomic analysis in this work.** (*A*) Pole-to-pole oscillation of the Min proteins establishes a concentration gradient of the division inhibition that is high at the poles (red shade), allowing formation of the FtsZ ring at the midcell. The cyan and green lines indicate the MinE and FtsZ rings, respectively. The chart shows the time-averaged protein concentration along the cell length. The red, cyan, and green lines represent the concentration of MinCD, MinE, and FtsZ, respectively. FtsZ is a tubulin homologue that assembles into a ring structure, the FtsZ ring, to recruit a series of septal proteins to the division site for the formation of the division septum. The FtsZ ring may also contribute constriction forces during cell division. (*B*) Micrographs showing GFP-MinD localization into a polar zone (red arrow) and MinE-GFP localization into a ring-like structure (blue arrow). (*C*) Experimental scheme. For clarity, only critical information is shown. The illustration of the intact cell is enlarged and is not in proportion to the size of the IM and OM vesicles.

The search for additional functions of the MinDE oscillator stemmed from knowledge of the partition function of the MinD/ParA family of proteins. Both MinD and ParA belong to a deviant form of the Walker-type ATPase family. Proteins in this family are reported to possess dynamic and filament-forming properties that could provide cytomotive force to mediate a range of macromolecular localization and targeting in bacteria (1). Examples include ParA of the P1 plasmid that actively segregates plasmids into daughter cells, MipZ of *Caulobacter crescentus* that localizes the divisome to the midcell, and PpfA of *Rhodobacter sphaeroides* that positions the chemotaxis machineries equidistance at opposite ends (reviewed in (10)). It appears that only one partition substrate associating with each system has been identified. There has been no systematic study to evaluate the global influence of these partition systems on cell physiology.

In this work, we took a quantitative proteomic approach, using the isobaric tags for relative and absolute quantitation (iTRAQ) method, to analyze the inner membrane (IM) proteome of the wild-type and Δ*min* strains, aiming to identify membrane proteins that are affected in the absence of the Min system. The iTRAQ data allowed us to identify 40 proteins of interest (POIs) that existed in differential abundance on the inner membrane of the Δ*min* mutant. POIs had two prominent features. First, 22 POIs were peripheral membrane proteins, which is also an inherent property of MinD and MinE in association with the membrane. Second, 18 POIs were identified as metabolic enzymes. We were able to identify the abundance change in metabolites that were correlated with the reactions catalyzed by POIs, suggesting that the Δ*min* cells are metabolically modulated. Meanwhile, we used the bacterial two-hybrid (B2H) assay and the pull-down assay to determine the protein–protein interaction (PPI). Six out of 10 POIs directly interacted with at least one of the Min proteins in both assays, which explained their identification as peripheral inner membrane (PIM) proteins. In searching the *E. coli* interactome data, we found no matches to the known PPIs involving the Min proteins in the databases. Thus, this study not only allows us to identify novel interactions of the Min system, but also reveals additional cellular functions that could be affected by the Min system.

## EXPERIMENTAL PROCEDURES

### 

#### 

##### Strains and Plasmids

The *E. coli* strains include wild-type MC1000 [Δ*lac* Δ*ara*] (11) and Δ*min* mutant YLS1 [*min::cat*] (12). For the B2H assay, pT25a and pT18a were modified from pT25 and pT18 (13), respectively, through several steps of PCR reactions. In brief, pT25a was engineered by removing the endogenous HindIII site (AAGCTT) in pT25, changing the BamHI site (GGATCC) in the multiple cloning site region to the SfoI site (GGCGCC), and introducing a new HindIII site adjacent to the SfoI site. The plasmid pT18a was created by removing the endogenous SfoI and BamHI sites in pT18, and then SfoI, HindIII, and BamHI sites were introduced upstream of the multiple cloning site. The target genes were PCR amplified from strain MC1000 and incorporated with a BamHI site before the stop codon that was followed by a HindIII site. An additional step was performed to introduce a silent mutation into *uspE* at Ile^72^ (atc to ata) to remove the internal BamHI site. The 5′-end of the PCR fragment was blunt-ended and the 3′-end was treated with HindIII for ligation into pT25a. The same PCR product was treated with BamHI at the 3′-end instead for ligation into pT18a.

For the pull-down assay, the plasmid encoding the bait protein included pSOT4 (P*_T7_::his_6_-t7-minD*), pSOT13 (P*_T7_::minE-his_6_*) (14), and pSOT80 (P*_T7_::malE-his_6_-minC*). The pHTPP plasmids were used in the construction of clones for protein overproduction. The plasmid pMLB1113 and its derivatives were used to generate clones carrying genes under an inducible *lac* promoter. To generate pSOT4, the *minD* fragment was PCR amplified to incorporate an EcoRI site (GAATTC) at the N terminus and an AvaI site (CYCGRG) after the stop codon, followed by restriction digestion and ligation into pHTPP13 (15). To generate pSOT80, the *minC* fragment was PCR amplified to incorporate an EcoRI site at the N terminus and an XhoI site (CTCGAG) after the stop codon, followed by restriction digestion and ligation into pHTPP14 (15). The green fluorescence protein (GFP) fusion protein was used as the prey that was expressed from the pMLB1113 derivatives (P*_lac_::gfp-gene* or P*_lac_::gene-gfp*). To create pMLB1113a, the endogenous SfoI site in pMLB1113 was inactivated by site-directed mutagenesis that introduced a silent mutation in *lacI*, followed by substituting the BamHI (GGATCC) site with the SfoI site within the multiple cloning site. The *gfp* gene with an upstream Shine–Dalgarno sequence was PCR amplified from pYLS49–2 and the EcoRI and SfoI sites were incorporated at the 5′- and 3′-ends of the DNA fragment, respectively. The DNA fragment was restriction digested and ligated into the corresponding sites in pMLB1113a to generate pMLB1113b. This plasmid allowed subsequent ligation of the target gene at the SfoI and HindIII sites to generate an N-terminal GFP fusion of the target protein. The plasmid pMLB1113c was created in two steps. The first step involved insertion of a DNA fragment (*GGATCC***T*AA****GCTT*CCT*GGATCC*) containing the BamHI-HindIII-BamHI sites in a consecutive order and an in-frame stop codon between the first BamHI and HindIII sites into the blunt-end treated SalI and HindIII sites in pMLB1113b. The 5′-end of *gfp* was modified through PCR that allowed generation of an in-frame fusion after digestion and religation with BamHI for cloning the target genes. The resulting plasmid was used as the template for amplification of the BamHI-HindIII-BamHI-*gfp* DNA fragment. A Shine–Dalgarno sequence followed by an SfoI site was introduced to the upstream of the DNA fragment in the second step. This PCR fragment was ligated into pMLB1113a at the EcoRI and XhoI sites to generate pMLB1113c. The plasmids used in the B2H and pull-down assays are summarized in Supplemental Table S1.

##### Isolation of Inner Membrane Fraction

One liter of cells cultured to the optical density (OD) of 0.6 at 600 nm was harvested, resuspended in 13 ml 10 mm Tris-Cl, pH8.0, 1 mm EDTA, 0.75 m sucrose, and incubated at room temperature for 10 min. Twenty-six milliliters of 10 mm Tris-Cl, pH8.0, 1 mm EDTA were added to dilute the sucrose. The cell suspension was then treated with 2 mg/ml lysozyme, 1 protease inhibitor tablet (Cat. 04 693 132 001, Roche, Basel, Switzerland), and 30 units DNase I (Cat. 89835, Thermo Fisher Scientific, Inc., MA, USA) at room temperature for 30 min. The concentration of KCl was brought to 150 mm before passing the suspension through a high-pressure cell disruption system (TS Series Cabinet, Constant System Ltd., Northants, UK) under 8,000 psi for three passages. The resulting lysate was centrifuged at 12,000 rpm for 30 min, and the supernatant was collected. This step was repeated once before the lysate was subjected to ultracentrifugation at 40,000 rpm (Optima l-90K centrifuge and SW45Ti rotor, Beckman Coulter, Inc., CA, USA) for 1 h. The pelleted membrane fraction was washed three times with 10 mm Tris-Cl, pH 7.6, 150 mm KCl, 1 mm EDTA, 1 mm DTT before resuspending in 1.6 ml 20% (w/v) sucrose solution prepared in the same buffer. The membrane suspension was laid on top of a two-step sucrose gradient that was made of 1.6 ml 70% (w/v) sucrose solution in the bottom layer and 4.8 ml 53% (w/v) sucrose solution in the upper layer prepared in an open-top ultracentrifugation tube (Cat. 355630, Beckman Coulter, Inc.). The tubes were placed in a fixed-angle rotor 70.1Ti (Beckman Coulter, Inc.), balanced, and centrifuged at 42,000 rpm at 25 °C for 16–18 h. After ultracentrifugation, we fractionated the gradient from the top every 0.5 ml. The NADH oxidase activity, which was highest in the inner membrane fraction, was determined by measuring absorbance at OD_340 nm_ caused by conversion of NADH to NAD. The protein concentration of each fraction was determined by the Bio-Rad Protein assay: Bradford (Cat. 500–0006, Bio-Rad Laboratories, Inc., CA, USA). The refractive index was run on a densitometer to confirm formation of a continuous gradient.

##### Labeling with Isobaric Tags for Relative and Absolute Quantitation (iTRAQ)

The inner membrane fraction containing 3 μg protein was run on an SDS-PAGE gel made of 4% stacking and 10% separation gels under 150V for 10–15 min. The gel was stained with 0.1% Coomassie blue solution for 10 min followed by destaining until a band of the total protein was visible. The band was excised and dissected into ∼1 mm^3^ cubes and transferred into a siliconized tube. The proteins in the gel slices were reduced, cysteine blocked, and digested through the treatment below. The gel slices were washed twice with 200 μl 50% acetonitrile (ACN)/25 mm ammonium bicarbonate (ABC), pH 8.5, and once with 200 μl 25 mm ABC, and incubated in 100 μl 50 mm dithioerythritol/25 mm ABC in the dark for 1 h at 37 °C. The buffer was removed and replaced with 100 μl 100 mm iodoacetamide (IAM)/25 mm ABC, and the reaction was incubated at room temperature in the dark for another hour. The gel slices were washed four times in 200 μl 50% ACN/25 mm ABC by vortex and incubated at room temperature for 15 min. The gel slices were dehydrated with 100% ACN before drying by SpeedVac. The dried sample was digested with trypsin prepared in 25 mm ABC at 37 °C overnight with a protein-to-trypsin ratio (w/w) of 10. The sample was then added with 50 μl 50% ACN/5% trifluoroacetic acid, sonicated, and centrifuged. The supernatant was transferred to a new tube and the steps of sonication and centrifugation were repeated two more times. The sample was dried using SpeedVac and dissolved in 10 μl 0.1% formic acid before clarification following a standard procedure of ZipTip C18 pipette tips (EMD Millipore, MA, USA). For quantitative proteomic analysis, digestion of proteins from each strain after enrichment was reacted with a different iTRAQ^®^ reagent (Roche) containing a different isobaric tag (114, 115, 116, or 117) following the manufacturer's instruction. Each individual labeled protein digest was purified through the ZipTip^®^ pipette tip before combining the protein samples of different strains for LC-MS/MS analysis. The protein digest of each strain from an independent culture was repeated four times, resulting in four sample repeats for analysis.

##### Mass Spectrometry for iTRAQ Analysis

NanoLC-nanoESI-MS/MS analysis was performed on a nanoAcquity system (Waters, MA, USA) connected to the Orbitrap Elite hybrid mass spectrometer (Thermo Electron, Bremen, Germany) equipped with a PicoView nanospray interface (New Objective, MA, USA). Peptide mixtures were loaded onto a 75 μm inner diameter, 25 cm length C18 BEH column (Waters) packed with 1.7 μm particles with a pore width of 130 Å and separated using a segmented gradient in 180 min from 5% to 40% solvent B (acetonitrile with 0.1% formic acid) at a flow rate of 300 nl/min and a column temperature of 35 °C. Solvent A was 0.1% formic acid in water. The mass spectrometer was operated in the data-dependent mode. Briefly, survey full scan MS spectra were acquired in the Orbitrap (*m/z* 350–1600) with the resolution set to 120K at *m/z* 400 and automatic gain control target at 10^6^. The 15 most intense ions were sequentially isolated for higher-energy collisional dissociation (HCD) MS/MS fragmentation and detection in the Orbitrap with previously selected ions dynamically excluded for 60 s. For MS/MS, we used a resolution of 15,000, an isolation window of 2 *m*/*z*, and a target value of 50,000 ions, with maximum accumulation times of 200 ms. Fragmentation was performed with normalized collision energy of 35% and an activation time of 0.1 ms. Ions with singly and unrecognized charge state were also excluded.

##### Protein Quantitation and Statistical Analysis

The mass spectrometry (MS) raw data were analyzed by the Proteome Discoverer^TM^ Software (v 1.4.1.14; Thermo Fisher Scientific, Inc.) for protein identification and iTRAQ 4plex quantitation. A database of *E. coli* strain K12 (organism ID 83333, 16,086 sequences) was downloaded from Uniprot (October 2013) and combined with the common Repository of Adventitious Proteins (116 sequences) from the Global Proteome Machine (http://www.thegpm.org) as the reference (16,202 entries in total) for initial analysis. Mascot (v 1.4; Matrix Science, MA, USA) was used for protein identification with the following settings: precursor mass tolerance window as 10 ppm, fragment tolerance window as 50 mmu, dynamic carbamidomethylation on cysteine, dynamic oxidation on methionine, dynamic N-terminal, and cysteine iTRAQ labeling. Two missed cleavages were permitted. All peptides of first rank in each spectrum with Mascot significant threshold less than 0.05 were used for data analysis to control the false discovery rate under 1% based on the target–decoy database algorithm.

The raw quantitation values of each iTRAQ tag for all peptides were exported from Proteome Discoverer and analyzed with an in-house statistical script using R language (http://www.r-project.org/). The script first integrated data from all four iTRAQ mass spectrometry experiments in which the four repeats in the experiments all had different tags (114, 115, 116, 117) to minimize any possible bias from iTRAQ labeling among experimental subjects, *i.e.* MC1000 and YLS1. Any peptides that were assigned to multiple protein groups were removed from the dataset. To normalize the system error among samples, the intensities of the peptides in YLS1 from each MS experiment were normalized by the log2 median ratios of YLS1 *versus* MC1000. To normalize the system error among different MS experiments, the normalized intensities of the peptides in each sample were normalized again by the ratio of total normalized tag intensity between the target MS experiment and a reference MS experiment (using the experiment with the highest total intensity as a reference). These normalized peptide intensities were then summed up to represent protein intensities per sample per MS experiment. To compare the difference of protein expression between two experimental subjects (YLS1 *versus* MC1000 with different isobaric labels), protein intensities were applied to log base 2 transformation and analyzed by paired-sample *t* test to examine if the mean difference between experimental subjects is different than 0. The mass spectrometry proteomics data have been deposited to the ProteomeXchange Consortium (16) via the PRoteomics IDEntifications (PRIDE) partner repository with the dataset identifier PXD002548.

Procedures for curating the reference library (Supplemental Table S2) and analyzing the IM proteome (Supplemental Table S3) can be found in Supplemental data.

##### Network Analysis

Network analysis was performed using Cytoscape (Version 3.1.0, supported by the National Institute of General Medical Sciences (NIGMS) and the National Resource for Network Biology (NRNB) ) (17, 18). The POI list was imported into Cytoscape to retrieve interaction data for building an initial network. The interaction data were downloaded from databases of the interactome browser mentha (1012 records), the complex portal IntAct (584 records), the Agile Protein Interaction DataAnalyzer (APID) (524 records), the Database of Interacting Proteins (DIP) (456 records), the automatic molecular interaction predictions Interoporc (132 records), BindingDB (25 records), European Bioinformatics Institute (EBI)-GO annotation (GOA)-IntAct (13 records), the Molecular INTeraction (MINT) (eight records), the Chemical database of EMBL (ChEMBL) (two records), and the Microbial Protein Interaction (MPI) (two records) that contained information on PPI, co-complex, and protein–ligand interaction. The initial network was filtered by mapping the entry identifiers (IDs) to the UniProt Reference Clusters, UniRef100, at a resolution that hides redundant sequences and obtains complete coverage of the sequence space. The protein entries were further filtered based on the reference library and combined with MS data. The resulting table was used to refine the protein network.

##### Metabolomic Analysis

Strains were grown in LB at 37 °C and harvested at the midexponential phase (OD_600 nm_ ∼0.6). Approximately 3 × 10^10^ cells were aliquoted into an Eppendorf tube, washed with 1.2 ml of ultrapure water five times, and the pellets were vacuum freeze-dried under a lyophilyzer. The dehydrated sample was then resuspended in ultrapure water to a density of 10^8^ colony forming units/μl and disrupted by sonication. The protein amount in each Eppendorf tube was quantified at this point. The resulting lysate was centrifuged at 14,000 rpm for 10 min to separate supernatant from the cell debris. The metabolites were extracted by mixing 150 μl supernatant with 450 μl of ACN by being kept at −20 °C for 16 h. After centrifugation at 14,000 rpm for 10 min, 400 μl of supernatant were vacuum dried and dissolved in 60 μl of ultrapure water. The dansylation step was performed by mixing 16 μl of sample with 2 μl of 0.5m carbonate buffer, pH 9.2, and 2 μl of 10 mg/ml dansyl chloride prepared in acetone, followed by incubation at 60 °C for 2 h. The reaction was terminated by the addition of 80 μl of ultrapure water and incubation at 60 °C for another 30 min. The supernatant of the reaction mixture after centrifugation was then subjected to liquid chromatography-electrospray ionization-tandem mass spectrometry (LC-ESI-MS) analysis of the positive ion mode. For aniline derivatization, 35 μl sample were mixed with 5 μl of 0.3 m aniline/HCl (molar ratio: 5/1) and 5 μl of 20 mg/ml 1-ethyl-3-(3-dimethylaminopropyl)-carbodiimide (EDC). The reaction was incubated at room temperature for 2 h and then halted with the addition of 5 μl of 10% ammonium hydroxide and incubation for 30 min. After centrifugation at 14,000 rpm for 10 min, the supernatant was subjected to LC-ESI-MS analysis of the negative ion mode.

The LC-ESI-MS system for metabolite analysis consisted of an ultraperformance liquid chromatography system (UltiMate 3000 RSLC, Dionex, Thermo Fisher Scientific, Inc.) and an electrospray ionization (ESI) source of quadrupole time-of-flight mass spectrometer (maXis HUR-QToF system, Bruker Daltonics, MA, USA). The autosampler was maintained at a constant temperature of 4 °C. The metabolites were separated by reversed-phase liquid chromatography on a BEH C18 column (2.1 × 100 mm, Waters). The sample was eluted with 99% mobile phase A (0.1% formic acid in deionized water) and 1% mobile phase B (0.1% formic acid in ACN), held at 1% B for 0.5 min, raised to 60% B in 5 min, further raised to 90% B in 0.5 min, held at 90% B for 1.5 min, and then lowered to 1% B in 0.5 min. The column was equilibrated by pumping 1% B for 4 min. The flow rate was set to 0.4 ml/min with injection volume 10 μl. LC-ESI-MS chromatogram was acquired using the following settings: capillary voltage of 4500 V in positive ion mode or 3500 V in negative ion mode, dry temperature at 190 °C, dry gas flow maintained at 8 l/min, nebulizer gas at 1.4 bar, and acquisition range of *m/z* 100–1,000. The acquired data were processed by the Target Analysis and Data Analysis software (Bruker Daltonics) that summarized an integrated area of signals. Found compounds were selected with the tolerance of LC peaks within 0.3 min and area higher than 200 counts from established compound identities.

The sample preparation was repeated six times for each strain and each preparation contained four replicates for MS analysis. The identified metabolite was matched to the *E. coli* Metabolome Database (ECMDB) (19). The measured counts were normalized with the cell dry weight that was determined by protein quantification to correct for variations between sample repeats. The probability to demonstrate the difference between metabolites isolated from different strains was calculated using the Student's *t*-test (two-tails, type 3). The ratio of the metabolite abundance between strains was calculated by subtracting the logarithmic number (base 2) of the dry-weight normalized intensity from the mutant with that from the wild type.

##### Bacterial Two-Hybrid Assay

Plasmids carrying gene fusions to the *cyaA* T25 or *cyaA* T18 fragment were cotransformed into an *E. coli* strain DHP1 [*F- glnV44(AS) recA1 endA1 gyrA96 (Nal^r^) thil hsdR17 spoT1 rfbD1*] (13). Fresh transformants were grown at 30 °C overnight in LB medium supplemented with 100 μg/ml ampicillin and 34 μg/ml chloramphenicol. 2.5 μl overnight culture were diluted to OD_600 nm_ 0.1 and spotted onto an LB plate containing 0.5 mm isopropyl β-d-1-thiogalactopyranoside, 40 μg/ml 5-bromo-4-chloro-3-indolyl-β-d-galactopyranoside (X-Gal), and antibiotics as described above. The plates were incubated at 30 °C to allow for color development.

##### Pull-Down Assay

The *E. coli* strains BL21(DE3) and BL21(DE3)/pLysS harboring plasmid pSOT4, pSOT13 (14), or pSOT80 were used to produce bait proteins, including MalE-His_6_-MinC, His_6_-MinD, and MinE-His_6_. Twenty-five milliliters overnight culture grown in LB containing 0.4% glucose and appropriate antibiotic were diluted into 1 l fresh LB containing supplements and grown at 30 °C until the OD_600_ was ∼0.6 before induction with 0.5 mm isopropyl β-d-1-thiogalactopyranoside. After a 3-h incubation, cells were harvested, resuspended in prechilled binding buffer (25 mm Tris-Cl, pH 8.0, 150 mm KCl, 5 mm imidazole) and broken using the cell disruption system as described earlier with settings of three passages at 20,000 psi at 4 °C. Crude lysate was collected and centrifuged at 20,000 rpm for 60 min at 4 °C. The clear lysate was mixed with 5 ml Ni Sepharose 6 Fast Flow resin (GE Healthcare Life Sciences, MA, USA) and incubated at 4 °C for 30 min with gentle inversion. The resin immobilized with the bait protein was then washed with 20 ml binding buffer for five times before use.

The POI tagged with GFP at the N- or C- terminus (Table S1), *i.e.* the prey, was expressed from the *E. coli* strain BL21(DE3). A 5 ml overnight culture was grown at 30 °C in LB supplemented with 0.4% glucose and 100 μg/ml ampicillin. The overnight culture was diluted into 5 ml fresh LB containing supplements and was allowed to grow until OD_600 nm_ reached 0.6. Protein expression was induced by 0.5 mm isopropyl β-d-1-thiogalactopyranoside for 3 h. Cells were then collected and washed twice with PBS (10 mm Na_2_HPO_4_, 1.8 mm KH_2_PO_4_, 137 mm NaCl, 2.7 mm KCl, pH 7.4). The pellet was resuspended in 200 μl PBS and lysed by 300 μl lysis buffer (B-PER Bacterial Protein Extraction Reagent, Thermo) containing 100 μg/ml lysozyme, 5U/ml DNase I, and protease inhibitor (Roche). The mixture was incubated at 4 °C for 60 min with gentle inversion. The clear lysate was obtained by centrifugation of the mixture at 13,000 rpm for 60 min at 4 °C. Four hundred microliters clear lysate containing the prey were mixed with 200 μl resin immobilized with the bait and incubated at 4 °C for 60 min with gentle inversion. The mixture was transferred into a 1 ml spin column and centrifuged at 800 rpm for 1 min at 4 °C. The column was washed five times by 500 μl binding buffer and another five times by 500 μl wash buffer (25 mm Tris-Cl, pH 8.0, 150 mm KCl, 30 mm imidazole). The bait and prey proteins were coeluted in 200 μl elution buffer (25 mm Tris-Cl, pH 8.0, 150 mm KCl, 300 mm imidazole). The eluted proteins were separated on 12% SDS-PAGE gels. The bait and prey were probed by Western blot analysis using anti-His_6_ (mouse anti-His antibody from mouse ascites fluid, GE Healthcare Life Sciences) and anti-GFP (GFP rabbit IgG polyclonal antibody fraction, Life Technologies, CA, USA) antibodies, respectively. In brief, the proteins were transferred from the gel onto a PVDF membrane. The membrane was blocked with 10% BSA [prepared in TBST (Tris-buffered saline 50 mm Tris-Cl, pH7.4, 150 mm NaCl, 0.1% tween20)] at 4 °C overnight. All antibodies were diluted in 5% BSA prepared in TBST. The membrane was incubated with the anti-His_6_ or anti-GFP antibody in 1:5000 dilution at room temperature for 1 h and washed three times with TBST. The membrane was then incubated with the secondary antibody conjugated with horseradish peroxidase, *i.e.* anti-mouse IgG antibody (goat anti mouse IgG (H+L), Jackson) or anti-rabbit IgG antibody (donkey anti rabbit IgG whole antibody, GE Healthcare), at 1:10,000 dilution for 1 h at room temperature. The signal was developed using the ECL kit (Amersham Biosciences^TM^ ECL^TM^ Select Western blotting Detection Reagent, GE Healthcare) and was detected on an ImageQuant LAS-4000 system (GE Healthcare Life Sciences).

## RESULTS

### 

#### 

##### Isolation and Characterization of the Inner Membrane Fraction

Because MinDE oscillates on the periphery of the inner membrane, we searched for the IM proteins that could be affected by the MinDE oscillator. The experimental procedure is outlined in Fig. 1*C*. The crude membrane of the wild-type and Δ*min* strains was isolated through sequential preparation and disruption of spheroplasts, followed by isopycnic sucrose-gradient ultracentrifugation to separate the inner membrane from the outer membrane (20, 21). A physiologically relevant concentration of KCl (150 mm) was added to the sucrose-gradient buffer to reduce nonspecific protein–membrane interaction. The IM fractions were characterized by elevated NADH oxidase activity (Supplemental Fig. S1), and the fraction with the highest NADH oxidase activity was used in the subsequent proteomic study.

##### Differential Abundance of the IM Proteins in the Δmin Mutant

The iTRAQ method allowed for multiplexing of different samples in the same run by MS analysis, which enables relative quantitation of IM proteins from different strains. Our experiments comprised four sample repeats each for both wild-type and Δ*min* strains in the iTRAQ labeling and MS analysis. The Pearson correlation coefficient, which represents the reproducibility between sample repeats, ranged from 0.90 to 0.95 for MC1000 and from 0.86 to 0.90 for YLS1 (Supplemental Table S3). A total of 45,759 peptides were identified and mapped to 865 unique proteins that were further curated to generate a subproteome containing 808 proteins based on the reference library of *E. coli* (Supplemental Table S2). We defined this subproteome as the IM proteome (Supplemental Table S3) hereafter. The MS intensity of proteins was converted by the log base 2 transformation before calculating the ratio that represented the difference in protein abundance between the mutant and wild-type membranes. We further set a cut-off ratio at ≥0.5 and a *p* value at ≤0.05 to obtain 40 POIs that showed a significant difference in abundance on the inner membrane of the mutant, among which, nine proteins had decreased abundance in the mutant (including MinD and MinE) and 31 had increased abundance in the mutant.

##### Subcellular Localization of IM Proteome Proteins and POIs

**Fig. 2.**
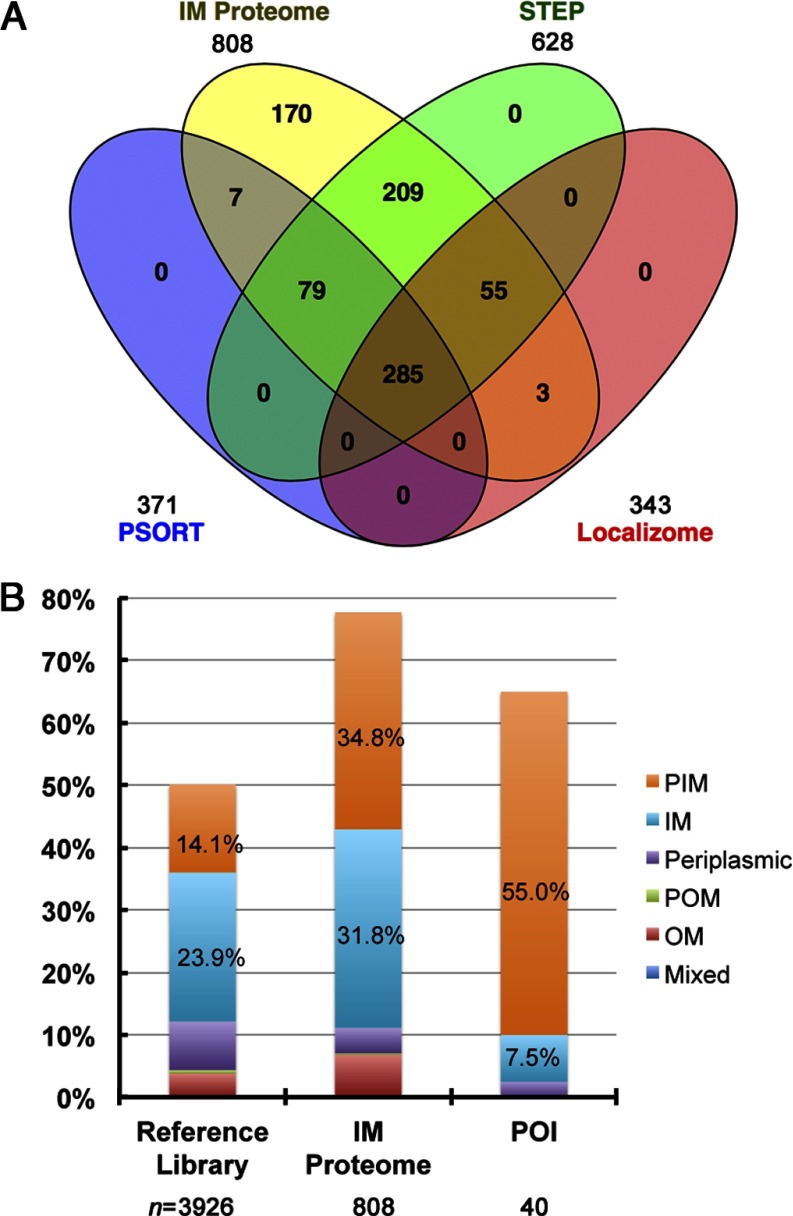
**Subcellular localization of the IM proteome proteins.** (*A*) A Venn diagram showing coverage of the IM proteome proteins in comparison with the membrane proteins documented in STEPdb, PSORTdb, and Dynamic Localizome. (*B*) Enrichment of PIM proteins demonstrated by comparison of subcellular localization of membrane proteins in reference library, IM proteome, and POI. The percentage of PIM and IM proteins is labeled in the chart. The number listed below each category is the population size (*n*).

Information about the IM proteome proteins (Supplemental Table S3) was extracted from the curated reference library of *E. coli* K-12, which contains subcellular localization data from databases of STEP (Subcellular Topologies of *E. coli* Polypeptides) (22, 23), PSORT (a database of protein subcellular localizations for bacteria and archaea) (24), ASKA clone (-) (A complete Set of *E. coli* K-12 ORF Archive; National BioResource Project, Japan), and Dynamic Localizome (25) (Supplemental data and Table S2). While subcellular localization described in STEPdb and PSORTdb concerns subcellular topology of proteins, subcellular localization documented in ASKA clone (-) and Dynamic Localizome emphasizes intracellular distribution of proteins. When the IM proteome was analyzed (Fig. 2A), the proteins identified to be common to STEPdb, PSORTdb, and Dynamic Localizome were mainly integral membrane proteins [75.4% (215/285)]. The common components found in both IM proteome and STEPdb were mainly peripheral membrane proteins [95.7% (200/209)]. Since the protein topology described in STEPdb and PSORTb is the most relevant for our purposes, we summarized the subcellular localization information only from the two databases as shown in Table I.

**Table I:** SCL terms of membrane proteins in E. coli

Location	Protein^a^	STEP	PSORT
Inner membrane (IM)	Peripheral IM (PIM)	Peripheral inner membrane protein facing the cytoplasm	
		Peripheral inner membrane protein facing the periplasm	
	
	IM	Integral inner membrane	Cytoplasmic membrane
		Inner membrane lipoprotein	

Periplasm	Periplasmic	Periplasmic	Periplasm

Outer membrane (OM)	Peripheral OM (POM)	Peripheral outer membrane protein facing the periplasm	
		Peripheral outer membrane protein facing the extracellular space	
	
	OM	Outer membrane β-barrel protein	Outer membrane
		Outer membrane lipoprotein	

^a^ Terms used in this study.

Meanwhile, 73.6% (595/808) of the IM proteome proteins were found to be membrane proteins. Among them, 90.4% (538/595) was located on the inner membrane. The analysis also demonstrated that the IM proteome was enriched in the PIM proteins (34.8%, 281/808) when compared with the reference library (Fig. 2*B*, Supplemental Table S3). More significantly, 55% (22/40) of the POIs were PIM proteins (Supplemental Table S4), pointing at the importance of the peripheral nature of proteins that we identified. This result also hinted that the Min system may regulate peripheral membrane interaction of proteins.

##### Enrichment of Metabolic Enzymes

**Fig. 3.**
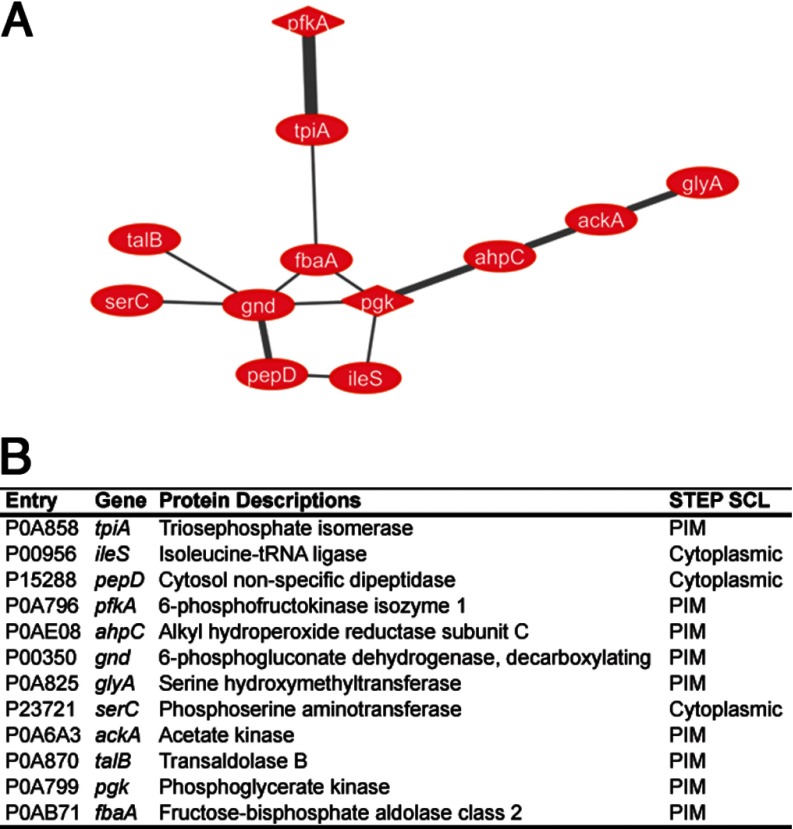
**Functional enrichment analysis of POI.** (*A*) A network view showing that 12 POIs are interconnected. The red shade (positive value) is applied to the nodes according to the ratio of protein abundance on the inner membrane. The thickness of the edges is drawn according to the mentha scores (34) that demonstrate evidence of PPI. (*B*) A table summarizing the node information in *A*.

We then focused on the 40 POIs for functional analysis and their interaction with the Min proteins. They were integrated into networks to visualize molecular interactions using the Cytoscape software platform (17, 18). In Cytoscape, genes are represented by “nodes” and interactions or other biological relationships are represented by “edges” between nodes. There were 470 nodes identified that connected some of the 808 IM proteome proteins, including all 40 POIs. Twelve out of the 40 POIs were connected directly or indirectly in a network (Fig. 3*A*), indicating their strong correlation. Interestingly, all 12 proteins are metabolic enzymes (hereafter metabolic POIs) that showed increased abundance on the inner membrane of the Δ*min* mutant, and eight proteins were annotated with a peripheral membrane location in STEPdb (Fig. 3*B*, Supplemental Table S4). The identification of various metabolic enzymes on the inner membrane of the Δ*min* mutant led us to perform metabolomics analysis in order to support the relevance of this observation to cellular metabolism.

##### Correlation between Metabolic POIs with Metabolites

To investigate metabolic correlation, we compared the metabolite profiles of the mutant *versus* the wild-type strain by LC-ESI-MS (Supplemental Table S5). The differential abundance of the metabolites was determined by the comparisons of the ratio of the peak intensity between the two strains. Among the 248 metabolites identified, 44 metabolites showed significant difference (*p* value<0.05) in the abundance between the wild-type and the mutant strain. Sorting the 44 metabolites based on the ECMDB classification, we found they were amino acids, peptides, and analogues, pyrimidine and purine nucleosides, and primary or secondary amines.

Next, we searched for whether any of the 44 metabolites are directly involved in the reactions catalyzed by metabolic POIs according to the EcoCyc database. Four POIs are found in five reactions that either utilize seven identified metabolites as the substrate or produce the identified metabolites (Table II). The POIs include (1) glycerol kinase (EC2.7.1.30) that catalyzes conversion of glycerol to *sn*-glycerol 3-phosphate in the glycerol degradation I pathway, (2) 3-phosphoserine aminotransferase (EC2.6.1.52) that catalyzes reversible conversion between phosphoserine with 2-oxoglutarate and 3-phospho-hydroxypyruvate with l-glutamate in the l-serine biosynthesis pathway, (3) aspartate aminotransferase, pyridoxal 5′-phosphate-dependent (aspartate aminotransferase, EC2.6.1.1) that catalyzes reversible conversion between l-aspartate with 2-oxoglutarate and oxaloacetate with l-glutamate in the l-aspartate biosynthesis and l-glutamate degradation II pathways, (4) tyrosine aminotransferase (aspartate aminotransferase, pyridoxal 5′-phosphate-dependent; EC2.6.1.5)that catalyzes reversible conversion between l-tyrosine with 2-oxoglutarate and 4-hydroxyphenylpyruvate with l-glutamate in the l-tyrosine biosynthesis I pathway, and (5) propionate kinase (acetate kinase, EC2.7.2.15) that catalyzes production of propionate from propanoyl phosphate in the l-threonine degradation I pathway. Among them, 3-phosphoserine aminotransferase and acetate kinase were found in the metabolic network of POIs (Fig. 3*B*). These observations further supported the correlation between the abundance change of the enzymes on the inner membrane and modulation of the enzymatic activity.

**Table II:** Metabolic enzymes and corresponding catalytic reactions. The information was retrieved from the EcoCyc database

Gene	Enzyme	EC No.	Pathway	Reaction^a^
*glpK*	glycerol kinase	2.7.1.30	glycerol degradation I	**glycerol** + ATP → *sn*-glycerol 3-phosphate + ADP + H^+^
*serC*	3-phosphoserine aminotransferase	2.6.1.52	l-serine biosynthesis	**3-phospho-l-serine** + 2-oxoglutarate ⇄ 3-phospho-hydroxypyruvate + **l-glutamate**
*aspC*	aspartate aminotransferase, PLP^b^-dependent	2.6.1.1	l-aspartate biosynthesis, l-glutamate degradation II	**l-aspartate** + 2-oxoglutarate ⇄ oxaloacetate + **l-glutamate**
*aspC*	tyrosine aminotransferase (PLP-dependent)	2.6.1.5	l-tyrosine biosynthesis I	**l-tyrosine** + 2-oxoglutarate ⇄ 4-hydroxyphenylpyruvate + **l-glutamate**
*ackA*	propionate kinase (acetate kinase)	2.7.2.15	l-threonine degradation I	ATP + propanoate ← ADP + propanoyl phosphate

^a^ Metabolite in bold face indicates that the abundance is increased in the mutant. Metabolite marked with underline indicates that the abundance is decreased in the mutant. Fold change: glycerol: 7.31; 3-phosph-l-serine: 2.69; l-glutamate: 1.97; l-aspartate: 2.1; l-tyrosine: 1.73; propanoate: 2.79.

^b^ PLP, pyridoxal 5'-phosphate.

It is worth noting that the metabolite abundance of polyamines, including putrescine, spermidine, and spermine, were significantly reduced in the mutant. Although enzymes of the spermidine biosynthetic pathway, such as spermidine synthase (SpeE) that catalyzes conversion of putrescine to spermidine, were not identified as POIs, they were detected in the IM proteome (Supplemental Table S3).

##### Interaction of POIs with MinC, MinD, and MinE

**Fig. 4.**
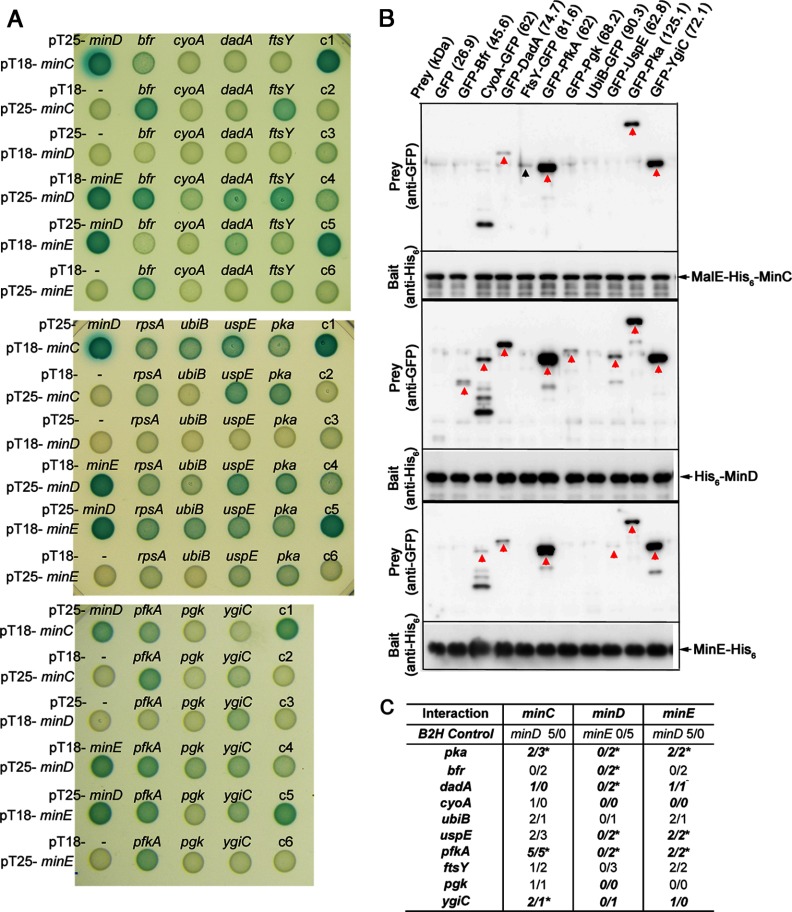
**Interaction between MinCDE and POIs.** (*A*) Direct interaction examined by the B2H assay. Interaction of *minC*, *minD*, and *minE* with *bfr*, *cyoA*, *dad*A, and *fts*Y (*upper panel*), *rpsA*, *ubiB*, *uspE*, and *pka* (*middle panel*), and *pfkA*, *pgk*, and *ygiC* (*lower panel*). RpsA, the 30S ribosomal subunit protein S1 was included as a control. The color was developed at 30 °C for 18 h before taking photographs. Control reactions: c1, pT25-*minD*/pT18-*minE*; c2, pT18-*minC*/pT25; c3, pT25-*minD*/pT18; c4, pT18-*minD*/pT25-*minD*; c5, pT25-*minE*/pT18-*minE*; c6, pT18-*minE*/pT25. (*B*) Direct interaction examined by the pull-down assay. The His-tagged MinC (*top*), MinD (*middle*), and MinE (*bottom*) were used to pull down the GFP-tagged POIs in the cell lysate. Each set contains two blots. The upper blot was hybridized with the anti-GFP antibody, and the lower blot was hybridized with the anti-His_6_ antibody. The red arrow indicates the signal detected at the expected molecular weight. The black arrow indicates the signal that was not at the expected molecular weight. (*C*) A summary of the interaction between MinCDE and POIs. The images were converted to 8-bit and the intensity of the colony was measured using ImageJ. The measurements were subtracted by the intensity of the corresponding negative control in each set of measurements. The scores represent the strength of intensity that was determined by the ratio of intensity between the examined pair of interaction and the positive control. The scoring criteria are as follows: 5—positive control as 100%; 4—greater than 50%; 3—30 to 50%; 2—10 to 30%; 1—less than 10% and regarded as the background. The two numbers are the scores of *minC*, *minD*, or *minE* when cloned in pT18 and pT25 for cross-examination. The interactions detected in the pull-down assays were shown in italics, bold letters. The asterisks indicated the reproducible interactions in both assays.

To investigate how the Min system affected the POIs, we examined PPI between them using the B2H assay. For this we selected 10 POIs, five with increased and five with decreased abundance in the mutant (Table III). The protein fusions were created at both N- and C-termini of the selected POIs and the Min proteins for cross-examination. Strikingly, positive interaction with at least one of the three Min proteins was detected at different degrees in eight selected POIs (Fig. 4*A*). Ubiquinol oxidase subunit 2 and phosphoglycerate kinase were the only clones that did not give clear signal above background in all combinations.

**Table III:** Information of POIs that were selected for verification

Protein	Protein Description	Ratio	*p* Value	STEP SCL[1]
**Pka**	Protein lysine acetyltransferase	-1.99	0.05	Cytoplasmic
**Bfr**	Bacterioferritin	-0.64	0.03	Cytoplasmic
**DadA**	d-amino acid dehydrogenase small subunit	-0.57	0.01	PIM
**CyoA**	Ubiquinol oxidase subunit 2	-0.53	0.03	IM
**UbiB**	Probable ubiquinone biosynthesis protein	-0.51	0.01	IM
**UspE**	Universal stress protein E	0.59	0.03	Cytoplasmic
**PfkA**	6-phosphofructokinase isozyme 1	0.59	0.02	PIM
**FtsY**	Signal recognition particle receptor	0.60	0.04	PIM
**Pgk**	Phosphoglycerate kinase	0.92	0.02	PIM
**YgiC**	Putative acid-amine ligase	0.96	0.01	Cytoplasmic

The pull-down assay was used to further verify the interaction. For this we fused the 10 POIs to GFP. We immobilized the purified MalE-His_6_-MinC, His_6_-MinD, or MinE-His_6_ on the Ni-NTA resin that was used to pull-down the GFP-tagged POI expressed in the crude cell lysate (Fig. 4*B*). Eight positive interactions with at least one of the three Min proteins were detected, but the result did not completely overlap with that of the B2H assay (Fig. 4*C*). For example, probable ubiquinone biosynthesis protein and signal recognition particle receptor were not detected in the pull-down assay. The consistent results between the two assays included interactions between protein lysine acetyltransferase and MinC/D/E, bacterioferritin and MinD, D-amino acid dehydrogenase small subunit and MinD, universal stress protein E and MinD/E, 6-phosphofructokinase isozyme 1 and MinC/D/E, and between putative acid-amine ligase and MinC. Phosphoglycerate kinase was the only protein that gave no convincing interaction in either assay. This could be due to the limitation of these assays, such as fusion tags of adenylate cyclase or GFP interfering with the interaction with the Min proteins. In addition, MinD was not functional when it was cloned in pT18 to introduce a CyaA' fragment at the C terminus of MinD. An alternative possibility is that changes in protein abundance on the inner membrane could have resulted from an indirect effect, such as interaction with an unidentified adaptor protein in the Min system. These possibilities could limit the detection power of these methods.

We searched the existing *E. coli* interactomes (26–28) to verify the existence of any novel interactions between the Min proteins and POIs (Supplemental Table S6). After excluding five interactions of MinC with the ribosomal subunits (27), we identified 49 interactions with the Min proteins in the interactomes, including 28 interactions with MinC, 13 interactions with MinD, and eight interactions with MinE (Supplemental Table S6). Among them, 17 interacting proteins (34.7%) were found in the IM proteome. Considering the significant statistical difference in protein abundance in the mutant inner membrane, three interactions involving FKBP-type peptidyl-prolyl cis-trans isomerase SlyD (with MinC), thioredoxin-1 TrxA (with MinD), and MinE (with MinD) were isolated. However, the fold change was low (1.15 fold for SlyD and 1.31 fold for TrxA, respectively) and they were not grouped into POI (1.41 fold and *p* value≤0.05 as the cut-off threshold). Additional MinD-interacting protein RNaseE, that was identified using the yeast two-hybrid system (29), did not show significant statistical difference between the two strains. In summary, this work identified novel direct interactions between POIs and MinCDE.

## DISCUSSION

We investigated the inner membrane proteome to search for any proteins that could be affected by the Min system. The quantitative proteomic approach allowed us to identify novel protein interactions with the Min system. In addition, the metabolic modulation accompanying the asymmetric division phenotype in the Δ*min* mutant revealed a physiological adaptation strategy from which the mutant could utilize the metabolic intermediates for synthesizing new materials presumably to rescue the abnormality of the mutant. The study further suggested that the function of macromolecule partition by the MinD/ParA family of the Walker-type ATPases may have broader roles in bacterial physiology.

**Fig. 5.**
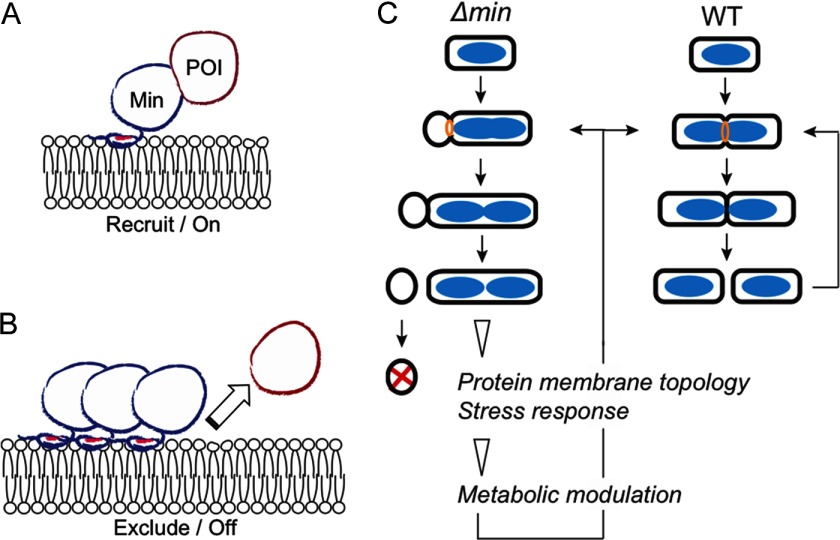
**Hypothetical models.** (*A*) The POI is recruited to the membrane proximity by MinC, MinD, or MinE (Min). It may subsequently interact with the inner membrane directly. (*B*) Exclusion of POI from the inner membrane by the Min proteins. (*C*) The aberrant division in the Δ*min* mutant shows a metabolic modulation phenotype that could be due to an imbalance between membrane-bound and cytosolic pools of the metabolic enzymes. Alternatively, uncharacterized stress responses could be involved in modulating the metabolic flux. Blue ovoid, nucleoid; orange circle, FtsZ ring.

We suggest the following models to explain the mechanism by which the Min system could influence the protein abundance on the inner membrane. First, the Min system may affect membrane localization of proteins through direct recruitment (Fig. 5*A*). Proteins that are actively recruited by the Min system to the inner membrane would decrease in abundance in the absence of the Min proteins. The examples include protein lysine acetyltransferase, bacterioferritin, D-amino acid dehydrogenase small subunit, and probable ubiquinone biosynthesis protein. In contrast, the Min system may exclude proteins from the inner membrane to facilitate protein recycling back to the cytosol. We would then expect an increased abundance of these proteins on the inner membrane of the mutant (Fig. 5*B*). The examples include universal stress protein E, 6-phosphofructokinase isozyme 1, signal recognition particle receptor, and putative acid-amine ligase. The aforementioned proteins showed direct interaction with at least one of the Min proteins in both the B2H and pull-down assays (Fig. 4), suggesting that both mechanisms involve direct interaction with the Min proteins. The purpose for the Min system-mediated localization of cytoplasmic proteins peripherally to the inner membrane could be to synchronize with cell growth and division via the Min oscillation to exploit the membrane environment for establishing their subcellular localization. On the other hand, the lack of any convincing interaction between the Min proteins and phosphoglycerate kinase (Fig. 4) suggests a third hypothetical mechanism by which the Min system could affect membrane localization of a protein through an adaptor protein that directly interacts with the Min system. It is also possible that some POIs were identified due to changes in the physicochemical property of the inner membrane relating to the lack of the Min system. Alternatively, some POIs were identified due to feedback of the stress response in the Δ*min* mutant, which could also affect overall abundance of POIs in a cell.

Based on our results, the Min system primarily affects PIM proteins (Fig. 2, Supplemental Table S4). The peripheral membrane interaction of a protein is reversible and can induce conformational change of a protein as well as modulate protein function in a spatiotemporally specific manner in eukaryotic cells (30, 31). The structural motifs that are known to support peripheral membrane localization include amphipathic helix, surface charged cluster of amino acids, and surface exposed hydrophobic patch (31). Given the fact that both MinD and MinE are PIM proteins that carry an amphipathic helix at the C- and N-termini, respectively, and the charge residues participate in membrane interaction of MinE (14, 32, 33), these structural features may be critical factors linking the membrane topology of POIs with the Min system (Supplemental data and Table S4).

An unexpected observation is a significant number of metabolic POIs functioning in various pathways (Fig. 3). Consistent with this observation, the metabolomic analysis identified metabolites that could be the substrate or produced in the reactions catalyzed by glycerol kinase, 3-phosphoserine aminotransferase, aspartate aminotransferase, and acetate kinase (Table II). This correlation strengthens a central idea of the work that the Min system could directly or indirectly modulate enzymatic activity by affecting protein targeting to the membrane location. Since the current analysis measures only accumulation of the metabolites instead of the metabolic flux, it is likely that more metabolic leads remain to be discovered.

Taken together, a balance between membrane-bound and cytosolic pools of the metabolic enzymes may be disturbed by the absence of the Min system, leading to a metabolic modulation phenotype in the Δ*min* mutant (Fig. 5*C*). The Δ*min* mutant allows division at the poles, leading to production of a chromosomeless minicell and a longer cell carrying multiple chromosomes. Minicells cannot proliferate, and multichromosomal cells could activate a rescue mechanism to rectify the abnormality. The aberrant division may also induce uncharacterized stress responses, causing a modulation in the metabolic flux. Therefore metabolic modulation is likely an adaptation strategy that a cell uses to overcome division defects in the absence of the Min system.

## Supplementary Material

Supplemental Data

## Footnotes

^1^ The abbreviations used are:

*E. coli*: *Escherichia coli*
WT: wild-type
*min*: minicelling
IM: inner membrane
PIM: peripheral inner membrane
POI: protein of interest
HPLC: high-performance liquid chromatography
MS: mass spectrometry
LC-MS/MS: liquid-chromatography tandem mass spectrometry
LC-ESI-MS: liquid chromatography-electrospray ionization-tandem mass spectrometry
iTRAQ: isobaric tags for relative and absolute quantitation
PPI: protein–protein interaction
B2H: bacterial two-hybrid
STEPdb: Subcellular Topologies of *E. coli* Polypeptides database
GFP: green fluorescence protein.
