# Supplementary material for: Quantitative Proteomics Analysis Reveals the Min System of Escherichia coli Modulates Reversible Protein Association with the Inner Membrane
Source: Mol Cell Proteomics. 2016 Feb 17;15(5):1572–83. doi: 10.1074/mcp.M115.053603 (PMC4858940; doi:10.1074/mcp.M115.053603)
Supplement: Supplemental Data [file supp_15_5_1572__index.html]

Quantitative proteomics analysis reveals the Min system of Escherichia coli modulates reversible protein association with the inner membrane — Quantitative Proteomics Analysis Reveals the Min System of Escherichia coli Modulates Reversible Protein Association with the Inner Membrane — Min System Modulates Peripheral Protein–Membrane Interaction — Supplemental Data 

# Quantitative Proteomics Analysis Reveals the Min System of *Escherichia coli* Modulates Reversible Protein Association with the Inner Membrane

## Supplemental Data

- Supplemental data (.pdf, 1.2 MB) - Supplemental data
- Table S3 (.xlsx, 11.9 MB) - Table S3 resubmission
- Table S4 (.xlsx, 49 KB) - Table S4 resubmission
- Table S5 (.xlsx, 509 KB) - Table S5 resubmission
- Table S6 (.xlsx, 58 KB) - Table S6 resubmission
