# Supplementary material for: Quantitative Proteomics Analysis Reveals the Min System of Escherichia coli Modulates Reversible Protein Association with the Inner Membrane
Source: Mol Cell Proteomics. 2016 Feb 17;15(5):1572–83. doi: 10.1074/mcp.M115.053603 (PMC4858940; doi:10.1074/mcp.M115.053603)
Supplement: Supplemental Data [file 10.1074_M115.053603_mcp.M115.053603-1.pdf]

Quantitative proteomics analysis reveals the Min system of *Escherichia coli* modulates reversible protein association with the inner membrane

Hsiao-Lin Lee (李曉苓)[1], I-Chen Chiang (江浥塵)[1], Suh-Yuen Liang (梁素雲)[1], Der-Yen Lee (李德彥)[2,4], Geen-Dong Chang (張震東)[2], Kwan-Yu Wang (王冠宇)[1], Su-Yu Lin (林淑妤)[1], Yu-Ling Shih (史有伶)[1,2,3*]

Supplemental Experimental Procedures

Reference library

The protein library of *E. coli* strain K12 (organism ID 83333) was retrieved from UniProtKB on February 16, 2015. The library was manually curated to exclude duplicates or disrupted proteins. In addition, the database of *E. coli* K-12 MG1655 obtained from EcoCyc (version 18.1) (1) and the reference tables of STEPdb based on *E. coli* BL21(DE3) (STEPdb 2.0 beta, http://www.stepdb.eu/step2/; downloaded on March 16, 2015) (2) were used to curate for pseudogenes and mobile elements, including genes from plasmid, phage, transposon, and Rhs elements. The operation resulted in a reference library of 3956 entries (Supplemental Table S2).

Protein subcellular localization

The subcellular localization (SCL) of proteins was obtained from four sources and combined into the reference library (Supplemental Table S2), including PSORTdb 2.0 based on the complete genome prediction of *E. coli* K12 MG1655 (downloaded on February 11, 2015) (3), STEPdb of *E. coli* BL21(DE3) (2, 4), ASKA clone (-) of *E. coli* K12 W3110 (National BioResource Project, Japan; downloaded on February 10, 2015), and Dynamic Localizome established using the ASKA clones (downloaded on February 7, 2015) (5).

In addition to the difference in *E. coli* strains, the SCL information was determined using different methodologies. STEPdb dissected subcellular topology of proteins using combinatorial methods of peripheral membrane proteome, bioinformatics, and reference searches, emphasizing the peripheral membrane proteins that were not addressed in other SCL databases (2). PSORTdb analyzed biological features that were known to influence or to be characteristic of the subcellular localization of proteins based on both experimental data and computational prediction (3). The ASKA clone dataset categorized the subcellular localizations of the C-terminal GFP fusion to proteins based on the structure and appearance of the fluorescence distribution (6). A general concern of the GFP fusion was to disrupt function and localization of a protein, which was reflected in a lower percentage of the membrane location in the dataset. Dynamic localizome quantitatively analyzed protein localization of the ASKA (-) clones

throughout the cell cycle, which provided a view of the dynamic protein localization over time (5).

Supplemental Results

Structural features that could support the peripheral membrane interaction of POIs

Because the peripheral membrane localization of MinD and MinE is critical for their oscillation behavior, we therefore analyzed structural features of the POIs that may be involved in the peripheral membrane interaction. Such structural features include amphipathic helix, protein surface charge, and hydrophobic loops (7, 8). We used the HeliQuest web tool (9) to analyze the terminal 50 residues at both the N- and C-termini of each POI with a window size of 18 amino acids to evaluate amphipathicity based on the predicted hydrophobic moment and the helical wheel projection. We then narrowed down the window size manually to verify the existence of the amphipathic helix of at least 2 helical turns at the protein termini. The isoelectric point (pI) of the terminal 15 and 5 residues at the C-terminus was calculated using the Compute pI/Mw tool under ExPASy, which is the SIB Bioinformatics Resource Portal. In this analysis, we considered a pI value to be highly charged if it was greater than or equal to 9 or less than or equal to 4. As shown in Supplemental Table S4, 72.5% (29/40) of the POIs were predicted to possess an amphipathic helix. Only 2 proteins (*dadA, gal*T) contained neither an amphipathic

helix nor a charged terminus under these criteria. The analysis suggested that amphipathic helices and charged clusters are common features of the POIs that could have a role in supporting the membrane interaction. The relevance of these features will require experimental verification.

This analysis was limited at the terminus regions due to the following reasons. First, it is not possible to predict a helix, a charged cluster, or a hydrophobic patch that is embedded inside a protein and only exposed under particular conditions. Second, a charged or hydrophobic patch in a protein does not have a definite size or a requirement for the continuity of amino acid residues. Third, the prediction could be affected if a POI forms a complex or in a ligand-bound form.

References

1. Keseler, I. M., Mackie, A., Peralta-Gil, M., Santos-Zavaleta, A., Gama-Castro, S., Bonavides-Martinez, C., Fulcher, C., Huerta, A. M., Kothari, A., Krummenacker, M., Latendresse, M., Muniz-Rascado, L., Ong, Q., Paley, S., Schroder, I., Shearer, A. G., Subhraveti, P., Travers, M., Weerasinghe, D., Weiss, V., Collado-Vides, J., Gunsalus, R. P., Paulsen, I., and Karp, P. D. (2013) EcoCyc: fusing model organism databases with systems biology. *Nucleic acids research* 41, D605-612

2. Orfanoudaki, G., and Economou, A. (2014) Proteome-wide sub-cellular topologies of E.coli polypeptides database (STEPdb). *Molecular & cellular proteomics : MCP*

3. Yu, N. Y., Laird, M. R., Spencer, C., and Brinkman, F. S. (2011) PSORTdb--an expanded, auto-updated, user-friendly protein subcellular localization database for Bacteria and Archaea. *Nucleic acids research* 39, D241-244

4. Papanastasiou, M., Orfanoudaki, G., Koukaki, M., Kountourakis, N., Sardis, M. F., Aivaliotis, M., Karamanou, S., and Economou, A. (2013) The Escherichia coli peripheral inner membrane proteome. *Molecular & cellular proteomics : MCP* 12, 599-610

5. Kuwada, N. J., Traxler, B., and Wiggins, P. A. (2015) Genome-scale quantitative characterization of bacterial protein localization dynamics throughout the cell cycle. *Molecular microbiology* 95, 64-79

6. Kitagawa, M., Ara, T., Arifuzzaman, M., Ioka-Nakamichi, T., Inamoto, E., Toyonaga, H., and Mori, H. (2005) Complete set of ORF clones of Escherichia coli ASKA library (a complete set of E. coli K-12 ORF archive): unique resources for biological research. *DNA research : an international journal for rapid publication of reports on genes and genomes* 12, 291-299

7. Whited, A. M., and Johs, A. (2015) The interactions of peripheral membrane proteins with biological membranes. *Chem Phys Lipids*

8. Cho, W., and Stahelin, R. V. (2005) Membrane-protein interactions in cell signaling and membrane trafficking. *Annu Rev Biophys Biomol Struct* 34, 119-151

9. Gautier, R., Douguet, D., Antonny, B., and Drin, G. (2008) HELIQUEST: a web server to screen sequences with specific alpha-helical properties. *Bioinformatics* 24, 2101-2102

10. Hsieh, C. W., Lin, T. Y., Lai, H. M., Lin, C. C., Hsieh, T. S., and Shih, Y. L. (2010) Direct MinE-membrane interaction contributes to the proper localization of MinDE in *E. coli*. *Molecular microbiology* 75, 499-512

Supplemental Tables

Table S1. Plasmid list.

Table S2. Reference library.

Sheet 1- Reference Library of *Escherichia coli*

Sheet 2- Pseudogene, interrupted gene, and mobile element (EcoCyc)

Sheet 3- Pseudogene, interrupted gene, and mobile element (Orfanoudaki et al., 2014)

Table S3. IM proteome.

Sheet 1- Peptide details (raw data)

Sheet 2- Peptides identified in the MS analysis

Sheet 3- Technical variation and reproducibility of the MS result

Sheet 4- Unique proteins

Sheet 5- IM Proteome proteins: Subcellular localization

Table S4. Proteins of interest.

Sheet 1- List of 40 POIs

Sheet 2- Prediction of structural features that may be involved in membrane interaction

Table S5. Metabolomic analysis.

Sheet 1- Raw data

Sheet 2- Raw data normalized with dry weight

Sheet 3- Data processing and statistical analysis

Sheet 4- Metabolites that showed significant difference in the mutant

Table S6. Min protein interactome

Sheet 1- Mining interacting proteins of MinCDE in the *E. coli* interactomes

Supplemental Figure legends

Fig. S1. Isolation and characterization of the inner membrane fractions from strains MC1000 (wild-type; A) and YLS1 (*Δmin*; B).

Fig. S2. An overview of the metabolic pathways showing metabolites and enzymes (italics) that were affected in the *Δmin* mutant.

Table S1. Plasmid list.

Gene \ Genotype	P_{lac}::*gene-cya'* (CyaA T18 domain)	P_{lac}::*cya"-gene* (CyaA T25 domain)	P_{lac}::*gfp-gene*	P_{lac}::*gene-gfp*
minC	pT18-minC	pT25-minC		
minD	pT18-minD	pT25-minD*		
minE	pT18-minE*	pT25-minE		
bfr	pSOT796	pSOT797	pSOT791	
cyoA	pSOT806	pSOT807		pSOY803
dadA	pSOT836	pSOT837	pSOT831	
ftsY	pSOT776	pSOT777		pSOT773
rpsA	pSOT826	pSOT827		
ubiB	pSOT816	pSOT817		pSOT813
uspE	pSOT786	pSOT787	pSOT781	
yfiQ	pSOT766	pSOT767	pSOT761	
pgk	pSOT646	pSOT647	pSOT641	
pfkA	pSOT896	pSOT897	pSOT891	
ygiC	pSOT886	pSOT887		pSOT883

* The plasmid was constructed in previous work (10).

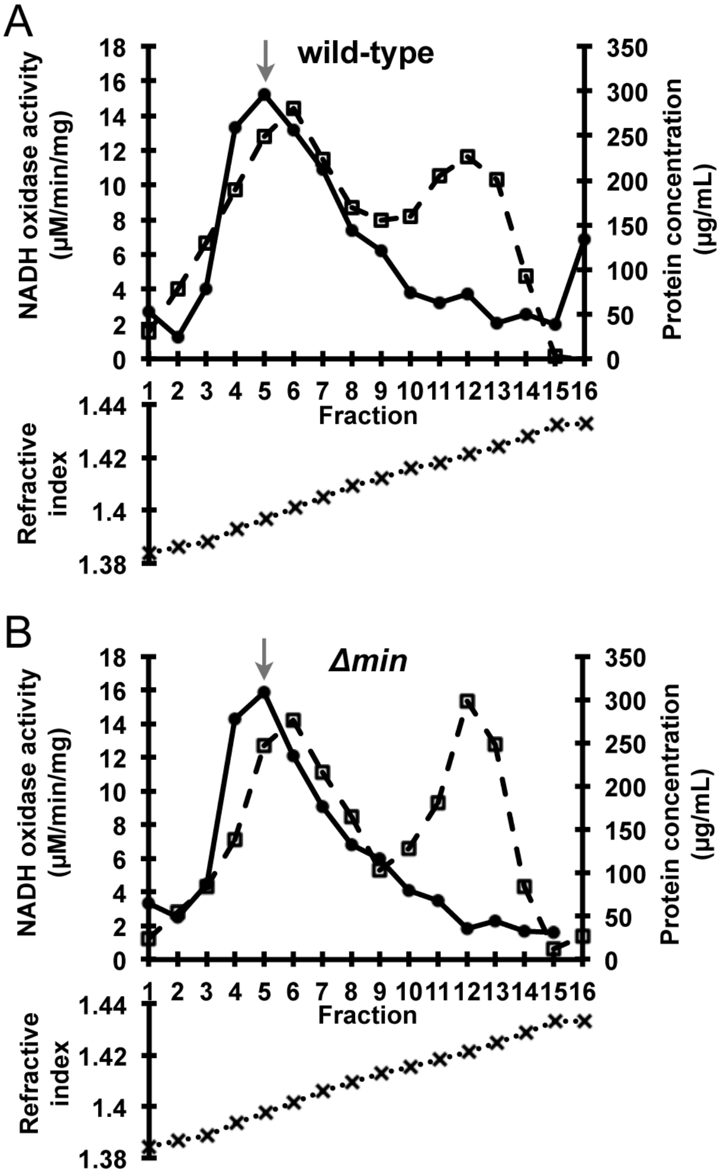

Fig. S1. Isolation and characterization of the inner membrane fractions from strains MC1000 (wild-type; A) and YLS1 (*Δmin*; B). The arrow indicates the peak fraction that was used for iTRAQ labeling and MS analysis.

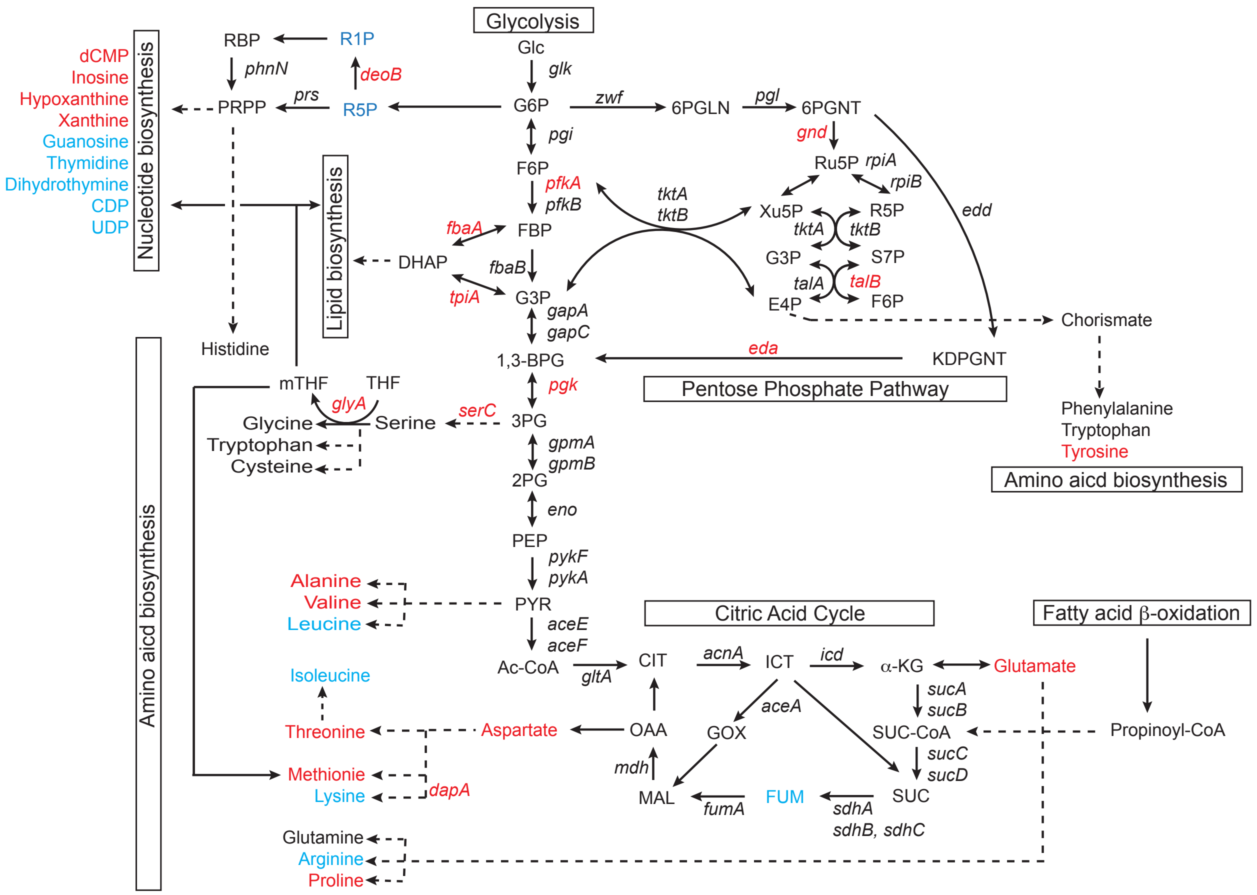

Fig. S2. An overview of the metabolic pathways showing metabolites and enzymes (italics) that were affected in the *Δmin* mutant. Red: increased abundance; blue: decreased abundance. Abbreviations: (1) Gene/Enzyme: Glk, glucokinase; Pgi, phosphoglucose isomerase; Pfk, 6-phosphofructokinase; Fba, FBP aldolase; TpiA, triose phosphate isomerase; GapA, GAP dehydrogenase; Pgk, phosphoglycerate kinase; Gpm, phosphoglycerate mutase; Eno, enolase; Pyk, pyruvate kinase; AceEF, pyruvate dehydrogenase; GltA, citrate synthase; AcnA, aconitate hydratase; Icd, isocitrate dehydrogenase; SucAB, SucCD, succinyl-CoA synthetase; SdhABC, succinate dehydrogenase; FumA, fumarase; Mdh, malate dehydrogenase; Zwf, glucose 6-

phosphate-1-dehydrogenase; Pgl, 6-phosphogluconolactonase; Edd, phosphogluconate dehydratase; RpiAB, ribose-5-phosphate isomerase; Gnd, 6-phosphogluconate dehydrogenase; TktAB, transketolase; TalAB transaldolase; Eda, Entner-Doudoroff aldolase; DeoB, phosphopentomutase; PhnN, ribose 1,5-bisphosphokinase; Prs, ribose-phosphate diphosphokinase; SerC, 3-phosphoserine aminotransferase; GlyA, serine hydroxymethyltransferase; DapA, 4-hydroxy-tetrahydrodipicolinate synthase; (2) Compound: Glc, glucose; G6P, glucose-6-phosphate; F6P, fructose-6-phosphate; PBP, fructose-1,6-biphosphate; DHAP, dihydroxyacetone phosphate; G3P, glyceraldehyde 3-phosphate; 1,3-BGP, 1,3-biphosphoglycerate; 3PG, 3-phosphoglycerate; 2PG, 2-phophoglycerate; PEP, phosphoenolpyruvate; PYR, pyruvate; 6PGLN, 6-phosphoglucono-δ-lactone; 6PGNT, 6-phophogluconate; Ru5P, ribulose-5-phosphate; R5P, ribose-5-phosphate; Xu5P, xylulose-5-phosphate; S7P, sedoheptulose-7-phosphate; E4P, erythrose-4-phosphate; Ac-CoA, acetyl coenzyme A; CIT, citrate; ICT, isocitrate; GOX, glyoxylate; α-KG, α-ketoglutarate; SUC-CoA, succinyl-coenzyme A, SUC, succinate; FUM, fumarate; MAL, malate; OAA, oxaloacetate; α-KG, α -ketoglutarate; G1P, glucose-1-phosphate; RBP, Ribose 1,5,-bisphosphate; R1P, α-D-ribose-1-phosphate; R5P, D-ribose 5-phosphate; PRPP, 5-phospho-α-D-ribose 1-diphosphate; THF, tetrahydrofolate; mTHF, 5,10-methylene-tetrahydrofolate.
